# Modified dementia risk score as a tool for the prediction of dementia: a prospective cohort study of 239745 participants

**DOI:** 10.1038/s41398-022-02269-2

**Published:** 2022-12-10

**Authors:** Zuo-Teng Wang, Yan Fu, Ya-Ru Zhang, Shi-Dong Chen, Shu-Yi Huang, Liu Yang, Hong-Qi Li, Ya-Nan Ou, Jian-Feng Feng, Qiang Dong, Wei Cheng, Lan Tan, Hui-Fu Wang, Jin-Tai Yu

**Affiliations:** 1 Department of Neurology, Qingdao Municipal Hospital, Qingdao University, Qingdao, China; 2 Department of Neurology and Institute of Neurology, Huashan Hospital, State Key Laboratory of Medical Neurobiology and MOE Frontiers Center for Brain Science, Shanghai Medical College, Fudan University, Shanghai, China; 3 Institute of Science and Technology for Brain-inspired Intelligence, Fudan University, Shanghai, China

**Keywords:** Diseases, Neuroscience

## Abstract

Based on risk profiles, several approaches for predicting dementia risk have been developed. Predicting the risk of dementia with accuracy is a significant clinical challenge. The goal was to create a modified dementia risk score (MDRS) based on a big sample size. A total of 239,745 participants from UK Biobank were studied (mean follow-up of 8.7 years). The score value of each risk factor was estimated according to the β coefficient in the logistic regression model. The total dementia risk score was the sum of each risk score. Kaplan Meier survival curves and Cox proportional hazards analyses were used to assess the associations between total score and dementia. Among all participants included, 3531 incident cases of all-cause dementia (ACD), 1729 cases of Alzheimer’s disease (AD), and 925 cases of vascular dementia (VD) were identified. Several vascular risk factors (physical activity, current smoking status, and glycemic status) and depressive symptoms were found to be significantly related to dementia risk. The modified dementia risk scores predicted dementia well (model 1, area under curve 0.810; model 2, area under curve 0.832). In model 1, the cut-off value for high risk (HR) was 81 or higher, and in model 2 (including the APOE4), it was 98 or higher. According to Kaplan-Meier survival analyses, patients in the HR group had faster clinical progression (*p* < 0.0001) in either model 1 or 2. Cox regression analyses for HR versus low risk (LR) revealed that the Hazard radio for ACD was 7.541 (6.941 to 8.193) in model 1 and 8.348 (7.727 to 9.019) in model 2. MDRS is appropriate for dementia primary prevention, and may help quickly identify individuals with elevated risk of dementia.

## Introduction

Dementia is a major and growing global health problem. There is currently no effective therapy available to treat dementia, emphasizing the importance of dementia prevention [1–3]. Early and accurate identification of people at high risk of dementia is critical for the effective implementation of preventive measures [4]. A clinically feasible risk assessment tool is necessary to identify high-risk individuals.

At present, several midlife risk scores are available to estimate dementia risk. The Cardiovascular Risk Factors, Aging and Dementia (CAIDE) risk score was designed to estimate dementia risk within 20 years [5]. The CAIDE score includes easily available midlife risk factors such as age, education, sex, systolic blood pressure, body-mass index, total cholesterol, physical inactivity, and *APOEε4* status, and has been validated in different populations [5, 6]. But the predictive capacity of CAIDE risk score is weak in several populations [7]. Tolea and colleagues tried to modify the CAIDE scoring system based on a small US population [8]. In addition, Schiepers and colleagues developed the Lifestyle for Brain Health (LIBRA) score based on the data from the Maastricht Ageing Study (MAAS) [9]. The Australian National University AD Risk Index (ANU-ADRI) is another self-report tool to identify dementia risk [10]. These two scores were based on small population sizes and may help in identifying risk status in dementia-prevention programmes in a short-term. Vascular risk factors for dementia are commonly used indicators in various scoring systems. In addition, depressive symptoms, as well as some lifestyles are also added to the risk scoring systems [9, 10]. Scoring systems incorporating *APOEε4* genotype, cerebrospinal fluid (CSF) biomarkers, blood-based biomarkers, or PET biomarkers may be more precise but not suitable for application in large-scale community studies [11].

The ideal risk assessment tool should be to use a few easily measurable risk factors that can be used to calculate the subsequent risk of dementia within a given time frame. We created dementia risk scores in this study by assessing several common risk factors using large-scale population data.

## Methods

### Study population

More than 500,000 participants, whose extensive phenotypic and genotypic data were collected at recruitment, of the UK Biobank (UKB) were recruited from 22 assessment centers across England, Scotland, and Wales between 2006 and 2010 [12]. Information on socio-demographics, habitual diet, lifestyle factors, and medical history was collected through touch-screen questionnaires; anthropometric data were obtained through physical measurements. Blood, urine, and saliva samples were also collected at baseline. All participants provided written informed consent. The UKB study has been approved by the North West Multi-Center Research Ethics Committee. In the present study, we included 239745 participants who had recorded data on health behavior, health status, lifestyle factors, and medical history (including age, education level, sex, physical activity level, smoking status, glucose level, Body mass index (BMI), Systolic blood pressure (SBP), total cholesterol level, depression status, *APOEε4* status, and follow-up time (at least one year)).

### Procedures

Age, sex, education, physical activity, current smoking status, and depressive symptoms data were collected at baseline as part of the UKB touchscreen questionnaire. Age was categorised into five groups (40–48, 49–55, 56–60, 60–64, and >64 years) based on quintiles (UKB Data-field ID: 21003). In UKB, the education qualifications were categorised into college or university degree; A levels, AS levels, or equivalent; O levels, GCSEs, or equivalent; CSEs or equivalent; NVQ, HND, HNC, or equivalent; other professional qualifications; none of the above (UKB Data-field ID: 6138). Participants were categorised into high (college or above), medium (High school or equivalent), and low (Less than high school) education level groups in this study [13]. Participants were asked “In a typical week, on how many days did you do 10 min or more of moderate physical activities like carrying light loads, cycling at normal pace? (Do not include walking)” (UKB Data-field ID: 884). Frequencies of physical activity consisted of 8 distinct values (0–7 days/week). Active people have leisure time physical activity at least one day/week; inactive people exercise less often than one day/week. The smoking status was categorised into current smoking and non-current smoking (UKB Data-field ID: 20116). Participants were asked “Over the past two weeks, how often have you felt down, depressed or hopeless?”. Participants were categorised into depressed and non-depressed groups (UKB Data-field ID: 2050). BMI value is constructed from height and weight measured during the initial assessment centre visit (UKB Data-field ID: 21001). The cut-off value of 30 kg/m^2^ was chosen for BMI [14]. SBP was read automatically by Omron device. Units of measurement are mmHg (UKB Data-field ID:4080). The cut-off value of 140 mm Hg was chosen for SBP [15]. Non-fasting plasma glucose was measured by hexokinase analysis on a Beckman Coulter AU5800 (UKB Data-field ID: 30740). The cut-off value of 11.1 mmol/L was chosen for glucose [16]. Total cholesterol was measured by CHO-POD analysis on a Beckman Coulter AU5800 (UKB Data-field ID: 30690).

### Ascertainment of dementia

According to the International Classification of Diseases (ICD), all-cause dementia (ACD) was defined as ICD-9 codes 290.2, 290.3, 290.4, 291.2, 294.1, 331.0, 331.1, 331.2, and 331.5, and ICD-10 codes A81.0, F00, F00.0, F00.1, F00.2, F00.9, F01, F01.0, F01.1, F01.2, F01.3, F01.8, F01.9, F02, F02.0, F02.1, F02.2, F02.3, F02.4, F02.8, F03, F05.1, F10.6, G30, G30.0, G30.1, G30.8, G30.9, G31.0, G31.1, G31.8, and I67.3 [17]. AD was defined as ICD-9 codes 331.0 and ICD-10 codes F00, F00.0, F00.1, F00.2, F00.9, G30, G30.0, G30.1, G30.8, and G30.9 [17]. VD was defined as ICD-9 codes 290.4 and ICD-10 codes F01, F01.0, F01.1, F01.2, F01.3, F01.8, F01.9, and I67.3 [17]. In addition, dementia diagnoses were also retrieved from primary care data using Read codes (Read v2 and Read v3). Detailed Read codes can be read in Additional Table 3.

### *APOE* genotyping

UKB participants were genotyped using two genotyping arrays manufactured by Affymetrix (the BiLEVE Axiom array and the UK Biobank Axiom array). Genotyping quality control was conducted by UKB centrally [18, 19]. Two genetic variants rs429358 and rs7412, were used to identify APOE ε2, ε3, and ε4 alleles. Participants with ε4 alleles were defined as APOE ε4 carriers.

### Statistical analysis

Demographic and baseline clinical characteristics were summarized using means, standard deviations (SDs), and proportions. Group comparisons in continuous variables were performed using Student’s t-tests. Chi-squared tests were used for categorical variables. Multiple logistic regression analyses were performed to examine the association between ACD and independent variables (SBP, BMI, total cholesterol, physical activity, current smoking status, glycemic status, and depressive status separately); all models were adjusted for age, sex, education status, and follow-up time. Independent variables significantly associated with dementia risk in the first analysis were included in the main logistic regression model (model 1). The main risk score analysis was performed by considering only the easily available measures. We then developed an additional risk score including APOE ε4 status into the model (model 2).

In addition, we built nomograms on the basis of these two logistic regression models. We assigned each factor an integer weight (0–100) according to the respective β coefficients. Individual risk scores were obtained by summing the scores for each risk factor [5]. To quantify the discrimination performance of the nomograms, Harrell’s C- indices were measured. The cut-off value for dementia risk score was thus determined by ROC curves. The pooled area under the AUC was calculated to determine the predictive performance of dementia risk scores. Kaplan-Meier survival analysis of clinical progression (progress to ACD, AD, and VD) was plotted based on the levels of dementia risk (low risk (LR) and high risk (HR)). Log-rank test was used to compare the survival distributions of the different levels of dementia risk. Cox proportional hazards models were used to test the predictive abilities of the dementia risk scores (continuous and grouping variables) for clinical progression. In addition, we performed the competing risk analysis to evaluate the impact of death. The “glm”, “pROC”, “survival”, “survminer”, “ResourceSelection”, “rms”, and “ggplot2” packages in R software (version 3.6.2) were used to perform the above analyses.

## Results

Of all 239745 individuals from UKB, 126361 were male (52.7%). They were aged between 40 to 73 years old at baseline. The mean follow-up time was 8.66 years (1–14 years), and 1.5% (3531) of participants were diagnosed with ACD. The differences between dementia and no dementia individuals are detailed in Table 1. Of all ACD individuals, 1729 (49.0%) were diagnosed with AD, and 925 (26.2%) were diagnosed with VD (Additional Table 2).

**Table 1:** Baseline demographic characteristics of participants included.

Characteristic	Non-demented (*n* = 236,214)	Demented (*n* = 3531)	*p*
Age (years, mean ± SD)	56.20 ± 7.96	64.25 ± 4.65	**<0.0001**
Education (high/intermediate/low, *n*)	89,791/96,272/50,151	1112/1510/909	**<0.0001**
Sex (male, %)	111,282 (52.89)	2102 (59.53)	**<0.0001**
Physical activity (active, %)	206,390 (87.37)	3087 (87.43)	0.9270
Current smoking status (yes, %)	22,021 (9.32)	340 (9.63)	0.5340
Glucose (mmol/L, mean ± SD)	5.09 ± 1.18	5.37 ± 1.54	**<0.0001**
BMI (kg/m², mean ± SD)	27.29 ± 4.70	27.22 ± 4.86	0.3750
SBP (mmHg, mean ± SD)	139.40 ± 19.40	145.35 ± 20.48	**<0.0001**
Total cholesterol (mmol/L, mean ± SD)	5.72 ± 1.13	5.51 ± 1.26	**<0.0001**
Depressive symptoms (yes, %)	53,564 (22.68)	865 (24.50)	**<0.0001**
APOE ε4 (yes, %)	67,301 (28.49)	1897 (53.72)	**<0.0001**
Follow-up time (years, mean ± SD)	8.67 ± 2.47	7.84 ± 2.56	**<0.0001**

Abbreviations: *BMI* body mass index, *AD* alzheimer disease, *SBP* systolic blood pressure, *DBP* diastolic blood pressure, *APOE* apolipoprotein E.

*p* values that are statistically significant are shown in bold.

Five variables (total cholesterol, physical activity, current smoking status, glycemic status, and depressive symptoms) significantly predicted dementia in the separate regression models (Additional Table 1). Given the inconsistency between cholesterol and dementia in previous studies, we did not include this variable in the final models [5, 20]. Physical activity, current smoking status, glycemic status, and depressive symptoms, together with age, education, sex, and follow-up time were put into regression model 1 simultaneously. Based on regression model 1, we further added APOE ε4 status in model 2. The scores assigned for factors have been assessed based on β coefficients (Table 2). The scores of some factors were changed after the addition of APOE ε4 status: the score for glycemic status decreased, and the scores for physical activity increased (Table 2).

**Table 2:** Logistic regression models for dementia risk, according to the risk factor profiles at middle age and the risk scores derived from the β coefficients.

Variables	Model 1				Model 2			
	β coefficient	*p*	OR (95% CI)	Score	β coefficient	*p*	OR (95% CI)	Score
Age								
40–48	0 (reference)			0	0 (reference)			0
49–55	1.0821	<0.0001	2.95 (2.13 to 4.16)	26	1.0842	<0.0001	2.96 (2.14 to 4.17)	26
56–60	2.2879	<0.0001	9.85 (7.34 to 13.55)	55	2.2893	<0.0001	9.87 (7.35 to 13.57)	55
60–64	3.1746	<0.0001	23.92 (17.99 to 32.64)	76	3.1890	<0.0001	24.26 (18.25 to 33.12)	76
>64	4.1733	<0.0001	64.93 (49.05 to 88.31)	100	4.1880	<0.0001	65.89 (49.77 to 89.63)	100
Education								
High	0 (reference)			0	0 (reference)			0
Intermediate	0.1849	<0.0001	1.20 (1.11 to 1.30)	4	0.1835	<0.0001	1.20 (1.11 to 1.30)	4
Low	0.2406	<0.0001	1.27 (1.16 to 1.39)	6	0.2400	<0.0001	1.27 (1.16 to 1.39)	6
Sex								
Women	0 (reference)			0	0 (reference)			0
Men	0.3974	<0.0001	1.49 (1.39 to 1.60)	10	0.3988	<0.0001	1.49 (1.39 to 1.60)	10
Physical activity								
Active	0 (reference)			0	0 (reference)			0
Inactive	0.1361	0.0092	1.15 (1.03 to 1.27)	3	0.1533	0.0036	1.17 (1.05 to 1.29)	4
Current smoking status								
No	0 (reference)			0	0 (reference)			0
Yes	0.2507	<0.0001	1.28 (1.14 to 1.44)	6	0.2445	<0.0001	1.28 (1.13 to 1.43)	6
Glycemic status								
≤11.1 mmol/L	0 (reference)			0	0 (reference)			0
>11.1 mmol/L	0.6903	<0.0001	1.99 (1.52 to 2.57)	17	0.6868	<0.0001	1.99 (1.51 to 2.57)	16
Depressive symptoms								
No	0 (reference)			0	0 (reference)			0
Yes	0.5081		1.66 (1.53 to 1.80)	12	0.5020		1.65 (1.52 to 1.79)	12
APOE ε4 status								
Non–ε4					0 (reference)			0
ε4					1.0996	<0.0001	3.00 (2.81 to 3.21)	26
Follow-up time	−0.1491	<0.0001			−0.1485	<0.0001		
Intercept	−6.3717	<0.0001			−6.8324	<0.0001		

Abbreviations: *APOE* apolipoprotein E, *CI* confidence interval, *OR* odds ratio.

**Fig. 1:**
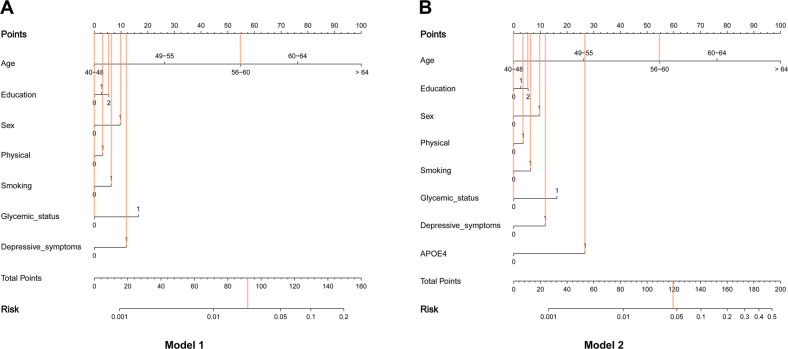
Nomogram models for the prediction of developing dementia. The value of each of variable was given a score on the point scale axis. A total score could be easily calculated by adding each single score. Corresponding markers for the total dementia risk scores and probability of dementia were shown in the nomograms. The probability of dementia increased as the dementia risk score increases. The patient was 59-years old men, and with low education, had low physical activity, in the current smoking status, had normal fasting blood glucose level (≤11.1 mmol/L), had depressive symptoms, and. carrying the ε4 alleles. Red lines are drawn upward to determine the points received by each variable; the sum (92 in model 1 (**A**), 119 in model 2 (**B**)) of these points is located on the Total Points axis, and a line is drawn downward to the risk axes to determine the probability of dementia (3.0% in model 1, 4.7% in model 2). APOE4 apolipoprotein E4.

**Fig. 2:**
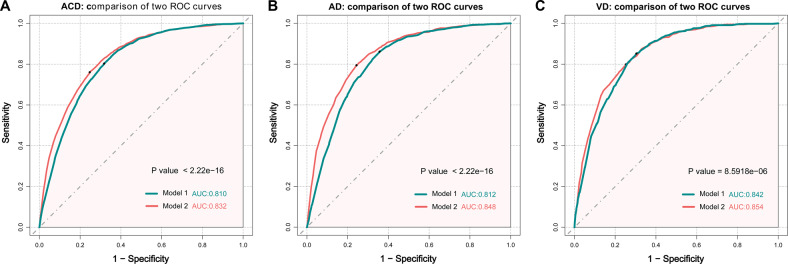
ROC curves show the performance of the dementia risk scores in predicting the risk of dementia. The AUC for model 1 was 0.810 (95% CI 0.804–0.816) for ACD (**A**). The AUC for model 1 was 0.812 (95% CI 0.804–0.820) for AD (**B**). The AUC for model 1 was 0.842 (95% CI 0.832–0.853) for VD (**C**). The AUC for model 2 was 0.832 (95% CI 0.826–0.838) for ACD. The AUC for model 2 was 0.848 (95% CI 0.840–0.856) for AD. The AUC for model 2 was 0.854 (95% CI 0.843–0.865) for VD. ACD all-caused dementia, AD alzheimer’s disease, VD vascular dementia, ROC receiver operating characteristic.

Moreover, corresponding markers for the total dementia risk scores and probability of dementia were shown in the nomograms (Fig. 1). The probability of dementia increased increases as the dementia risk score increases (Fig. 1). In addition, Fig. 1 shows an example of using the nomogram to predict the probability of dementia of a given patient. The Harrell’s C-index for the prediction nomogram was 0.810 for model 1, and 0.832 for model 2. The receiver operating characteristic analysis depicts the predictive potential of dementia risk score for subsequent dementia. In the basic model 1 risk score, the best cut-off was identified to be the score value of 81 points or more for ACD (AUC = 0.810, *p* < 2.22E-16, Sensitivity = 0.803, Specificity = 0.682), the best cut-off was identified to be the score value of 79 points or more for AD (AUC = 0.812, *p* < 2.22E-16, Sensitivity = 0.861, Specificity = 0.643), and the best cut-off was identified to be the score value of 83 points or more for VD (AUC = 0.842, *p* < 8.59E-6, Sensitivity = 0.852, Specificity = 0.696). In the model 2, the best cut-off was identified to be the score value of 98 points or more for ACD (AUC = 0.832, Sensitivity = 0.760, Specificity = 0.753), the best cut-off was identified to be the score value of 100 points or more for AD (AUC = 0.848, Sensitivity = 0.794, Specificity = 0.757), and the best cut-off was identified to be the score value of 98 points or more for VD (AUC = 0.854, Sensitivity = 0.799, Specificity = 0.747) (Fig. 2). We selected the cut-off value for HR as 81 or more in model 1 and 98 or more in model 2.

**Fig. 3:**
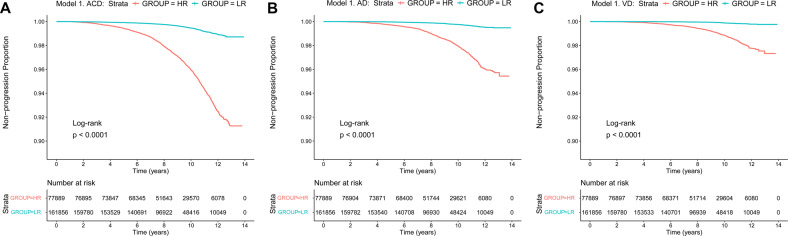
Kaplan**–**Meier survival curves predict ACD, AD, and VD risk in the basis of model 1. Patients in the HR group had faster clinical progression (*p*logrank  <  0.0001) compared with those in the LR group in model 1 for ACD (**A**), AD (**B**), and VD (**C**). ACD all-caused dementia, AD Alzheimer’s disease, VD vascular dementia, LR low risk, HR high risk.

**Fig. 4:**
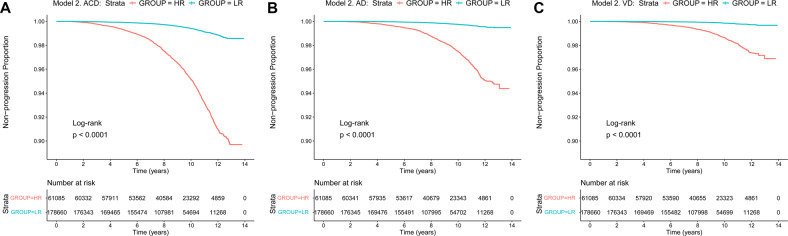
Kaplan**–**Meier survival curves predict ACD, AD, and VD risk in the basis of model 2. Patients in the HR group had faster clinical progression (*p*logrank  <  0.0001) compared with those in the LR group in model 2 for ACD (**A**), AD (**B**), and VD (**C**). ACD all-caused dementia, AD alzheimer’s disease, VD vascular dementia, LR low risk, HR high risk.

Kaplan-Meier survival analyses suggested that patients in the HR group had faster clinical progression (plogrank < 0.0001) compared with those in the LR group, either in model 1 or in model 2 (Figs. 3 and 4). Cox regression analyses suggested that the dementia risk scores were strong prognostic indicators for ACD, AD and VD in these two models. In the model 1, the hazard ratios for ACD were 1.039 (continuous) and 7.541 (HR vs. LR), the hazard ratios for AD were 1.039 (continuous) and 8.595 (HR vs. LR), and the hazard ratios for VD were 1.049 (continuous) and 11.810 (HR vs. LR) (Table 3). In the model 2, the hazard ratios for ACD were 1.040 (continuous) and 8.348 (HR vs. LR), the hazard ratios for AD were 1.044 (continuous) and 10.550 (HR vs. LR), and the hazard ratios for VD were 1.045 (continuous) and 10.400 (HR vs. LR) (Table 3). In the competing risk analyses, patients in HR groups had higher dementia risk compared to the LR groups when considering death as competing risk (*p* < 0.0001) (Additional Fig. 1 and Additional Fig. 2).

**Table 3:** Cox regression analysis on the dementia risk score associated with dementia subgroups.

Variable	ACD		AD		VD	
	Hazard ratio (95% CI)	*P* value	Hazard ratio (95% CI)	*P* value	Hazard ratio (95% CI)	*P* value
Model 1						
Dementia risk score (continuous)	1.039 (1.037 to 1.040)	<0.0001	1.039 (1.037 to 1.042)	<0.0001	1.049 (1.046 to 1.052)	<0.0001
Dementia risk score (HR vs. LR)	7.541 (6.941 to 8.193)	<0.0001	8.595 (7.596 to 9.725)	<0.0001	11.810 (9.783 to 14.270)	<0.0001
Model 2						
Dementia risk score (continuous)	1.040 (1.038 to 1.041)	<0.0001	1.044 (1.042 to 1.046)	<0.0001	1.045 (1.043 to 1.048)	<0.0001
Dementia risk score (HR vs. LR)	8.348 (7.727 to 9.019)	<0.0001	10.550 (9.378 to 11.880)	<0.0001	10.400 (8.856 to 12.220)	<0.0001

Abbreviations: *ACD* all-cause dementia, *AD* Alzheimer’s disease, *VD* vascular dementia, *HR* high risk, *LR* low risk.

## Discussion

This study was designed to develop modified dementia risk scores (MDRS) based on a large-scale dataset that can be easily used for assessing dementia risk. Previous studies have reported multiple modifiable and non-modifiable risk factors for dementia, and this study was also conducted based on these factors [21, 22]. Our study suggested that the MDRS can provide good predictions of dementia within 1–14 years. The MDRS can also predict the future risk of AD and VD well. More research is needed regarding the validity and transferability of this MDRS.

Previous researches suggested that the primary prevention of dementia was important [23]. The early identification of individuals at risk for dementia may contribute to the development of preventive strategies. Multiple biomarkers may serve as predictors of dementia in healthy adults [24–29]. However, most of the biomarkers are not readily available in clinical practice. Therefore, several brief dementia screening tools have been developed using easily available risk factors [5, 30–33]. Among them, the CAIDE score was an efficacious dementia-risk score system, which can predict the later risk of dementia on the basis of the midlife risk factors [5]. Increasing age is the strongest known risk factor for dementia [22, 34]. In our study, age accounted for a large proportion of the MDRS. In fact, previous study also show the importance of age in the predictive performance of CAIDE score [35]. The age was categorised into three groups based on tertiles in CAIDE. We changed the age groups based on quintiles (categorised into five groups). This MDRS focuses more on the role of age compared to the CAIDE score. The age is likely to be an important driver of the MDRS predictive ability. The UKB participants were younger at baseline, and the follow-up time of the UKB participants was shorter than that in the CAIDE study. These may partially explain the differences between the two scoring systems.

It has been reported that the increased level of cholesterol was a risk factor for dementia [36–38]. The higher level of total cholesterol was part of the CAIDE scoring system. However, several evidences indicate that, in some cases, an increased level of cholesterol was associated with decreased risk of dementia and slower cognitive decline [20, 39, 40]. The effect of cholesterol on dementia may be modified by vascular risk factors [20]. Malik and colleagues’ study using UKB data to study the relationship between midlife vascular risk factors and dementia risk and found that baseline low-density lipoprotein cholesterol (LDL-C) levels were significantly higher in no dementia populations than in dementia populations, which was consistent with our results [41]. Therefore, the total cholesterol was not incorporated into our scoring models. Furthermore, compared to the CAIDE score, we did not include BMI in the final models. Controversies persist regarding the relationships between BMI and the risk of dementia. These relationships may be modified by age, and such a dichotomy may not embody the effect of BMI on dementia [42]. In addition, we didn’t find significant associations between BMI and dementia in logistic regression analyses. Compared to the CAIDE score, we additionally included current smoking status and depressive status in the final models. Current smoking and depressive symptom are modifiable risk factors for dementia, which can be surveyed easily [22, 43–46]. Regardless of the models for dementia risk scores, the AUC values were greater than 0.8 (between 0.810 and 0.854) in our study. This implied that the MDRS performed well in predicting these multifactorial diseases (including ACD, AD, and VD). The modified score values, like the CAIDE score, were also derived from β coefficients. The integer scoring system was more convenient for clinical applications.

The modified risk scores have several strengths. First, the modified risk scores were based on big population data. We included 239745 UKB individuals in this study, which ensured sufficient statistical power. Another strength was the use of two models. One model contains the *APOE4* genotype (model 2), while another does not contain the *APOE4*. Although the model with *APOE4* was more accurate in predicting dementia, applying model without *APOE4* can still well predict dementia risk under some conditions where genotyping data are not available. Furthermore, we also validated the two scoring systems using Cox regression models. Several limitations may be pointed out. First, most of the variables data were obtained from self-reported questionnaire surveys, which were susceptible to some bias in responses. Second, the UKB participants were predominantly white, and we did not explore the diversity among different ethnic groups. Third, our scoring system may be more appropriate to predict the dementia risk in a relatively short time frame due to the limited follow-up time (mean 8.66 years). Fourth, we have not incorporated additional risk factors for dementia into the models. The inclusion of more risk factors in future models may help to improve prediction accuracy. The accuracy can be further improved in studies including more risk factors and cohorts with long-term follow-up.

These modified risk scores are appropriate for dementia primary prevention. We hope to identify patients with elevated risk of dementia quickly. Of the risk factors included in the modified dementia scoring systems, several vascular risk factors (physical activity, current smoking status, and glycemic status) are within intervention scope. Dementia incidence might be reduced by reducing vascular risk factors and improving depressive symptoms. Further studies are needed to validate the efficacy of these modified risk scores.

## Supplementary information

Supplemental Information

## Data Availability

The datasets described in this manuscript are available from the UK Biobank with an approved protocol. External investigators can request the data and approval of use on application to the UK Biobank (www.ukbiobank.ac.uk/).
