# Supplementary material for: Modified dementia risk score as a tool for the prediction of dementia: a prospective cohort study of 239745 participants
Source: Transl Psychiatry. 2022 Dec 10;12:509. doi: 10.1038/s41398-022-02269-2 (PMC9741578; doi:10.1038/s41398-022-02269-2)

| **Additional table 1. Logistic regression models for dementia risk, according to the risk factor profiles.** | | | |
| --- | --- | --- | --- |
| **Variables** | **β coefficient** | **p** | **OR (95% CI)** |
| **Systolic blood pressure** |  |  |  |
| >140 mm Hg vs ≤140 mm Hg | -0.0311 | 0.3910 | 0.97 (0.90 to 1.04) |
| **Body-mass index** |  |  |  |
| >30 kg/m² vs ≤30 kg/m² | 0.0195 | 0.6360 | 1.02 (0.94 to 1.10) |
| **Total cholesterol** |  |  |  |
| >6·5 mmol/L vs ≤6·5 mmol/L | -0.1951 | < 0.0001 | 0.75 (0.69 to 0.90) |
| **Physical activity** |  |  |  |
| Inactive vs Active | 0.1729 | 0.0009 | 1.19 (1.07 to 1.31) |
| **Current smoking status** |  |  |  |
| Yes vs No | 0.2865 | < 0.0001 | 1.18 (1.18 to 1.49) |
| **Glycemic status** |  |  |  |
| >11.1 mmol/L vs ≤11.1 mmol/L | 0.7261 | < 0.0001 | 2.07 (1.57 to 2.67) |
| **Depressive symptoms** |  |  |  |
| Yes vs No | 0.5223 | < 0.0001 | 1.69 (1.56 to 1.82) |
| NOTE. were first included separately in a logistic regression model together with age, sex, education, and follow-up time. Active people have leisure time physical activity at least 1 days/week; inactive people exercise less often than 1 days/week. Abbreviations: APOE, apolipoprotein E. | | | |

| **Additional table 2. Baseline demographic characteristics of the demented participants** | | | |
| --- | --- | --- | --- |
| Characteristic | ACD (n=3531) | AD (n=1729) | VD (n=925) |
| Age (years, mean ± SD) | 64.25 ± 4.65 | 64.52 ± 4.31 | 64.94 ± 4.17 |
| Education ( high/intermediate/low, n) | 1112/1510/909 | 532/755/442 | 211/427/287 |
| Sex (male, %) | 2102 (59.5) | 942 (54.5) | 596 (65.5) |
| Physical activity (active, %) | 3087 (87.4) | 1538 (89.0) | 821 (88.8) |
| Current smoking status (yes, %) | 340 (9.6) | 130 (7.5) | 112 (12.1) |
| Glucose (mmol/L, mean ± SD) | 5.37 ± 1.54 | 5.25 ± 1.31 | 5.62 ± 1.99 |
| BMI (kg/m², mean ± SD) | 27.22 ± 4.86 | 26.91 ± 4.65 | 27.69 ± 5.17 |
| SBP (mmHg, mean ± SD) | 145.35 ± 20.48 | 145.55 ± 20.20 | 147.37 ± 21.65 |
| Total cholesterol (mmol/L, mean ± SD) | 5.51 ± 1.26 | 5.61 ± 1.24 | 5.40 ± 1.35 |
| Depressive symptoms (yes, %) | 865 (24.5) | 388 (22.4) | 229 (24.8) |
| APOE ε4 (yes, %) | 1897 (53.7) | 1085 (62.8) | 465 (50.3) |
| Follow-up time (years, mean ± SD) | 7.84 ± 2.57 | 7.94 ± 2.46 | 7.98± 2.49 |
| Abbreviations: BMI, body mass index; ACD, all-cause dementia; AD, Alzheimer disease; SBP, systolic blood pressure; DBP, diastolic blood pressure; APOE, apolipoprotein E. | | | |

| **Additional table 3. Read codes used for dementia diagnosis and classification** | | |
| --- | --- | --- |
| **Diagnosis** | **Code Type** | **Codes** |
| ACD | Read V2 | 1461, A411., A4110, E00.., E000., E001., E0010, E0011, E0012, E0013, E001z, E002., E0020, E0021, E002z, E003., E004., E0040, E0041, E0042, E0043, E004z, E012., E02y1, E041., Eu00., Eu000, Eu001, Eu002, Eu00z, Eu01., Eu010, Eu011, Eu012, Eu013, Eu01y, Eu01z, Eu02., Eu020, Eu021, Eu022, Eu023, Eu024, Eu025, Eu02y, Eu02z, Eu041, Eu106, Eu107, F110., F1100, F1101, F111., F112., F116., F118., F11x2, F11x7, F11x9, F11y2, F21y2, Fyu30, 38C13, 3AE3., 3AE4., 3AE5., 3AE6., 66h.., 6AB.., 8BM02, 8BM50, 8BM60, 8BPa., 8CMe0, 8CMG2, 8CMZ., 8CMZ0, 8CMZ1, 8CMZ2, 8CMZ3, 8CSA., 8Hla., 8IAe0, 8IAe2, 9hD.., 9hD0., 9hD1., 9Ou.., 9Ou1., 9Ou2., 9Ou3., 9Ou4., 9Ou5. |
|  | Read CTV3 | .1461, 1461, .E11., .E111, .E112, .E113, .E114, .E115, .E116, .E11Z, .F21Z, .F371, .G78., A411., A4110, E00.., E000., E001., E0010, E0011, E0012, E0013, E001z, E002., E0020, E0021, E002z, E003., E004., E0040, E0041, E0042, E0043, E004z, E012., E02y1, E041., Eu00., Eu000, Eu001, Eu002, Eu00z, Eu01., Eu010, Eu011, Eu012, Eu013, Eu01y, Eu01z, Eu02., Eu020, Eu021, Eu022, Eu023, Eu024, Eu025, Eu02y, Eu02z, Eu041, F110., F1100, F1101, F111., F112., F116., F118., F11x2, F11x7, F11y2, F21y2, Fyu30, Ub1T6, X002m, X002w, X002x, X002y, X002z, X0030, X0031, X0032, X0033, X0034, X0035, X0036, X0037, X0039, X003A, X003B, X003C, X003D, X003E, X003F, X003G, X003H, X003I, X003J, X003l, X003m, X003P, X003R, X003T, X003V, X003W, X003X, X00R2, X00Rk, Xa0lH, Xa0sC, Xa0sE, Xa1GB, Xa25J, Xa3ez, XaA1S, XabVp, XaE74, XaIKB, XaIKC, XaKyY, XaOfZ, XE17j, XE1aG, XE1Xs, XE1Xu, XE1Z6, .3AE3, .3AE4, .3AE5, .3AE6, .66h., .6AB., .9hD1, .9Ou., .9Ou1, .9Ou2, .9Ou3, .9Ou4, .9Ou5, 3AE3., 3AE4., 3AE5., 3AE6., 66h.., 6AB.., 8BM02, 8BM50, 8BPa., 8CMe0, 8CMG2, 8CMZ., 8CMZ0, 8CMZ1, 8CMZ2, 8CMZ3, 8CSA., 8IAe0, 8IAe2, 9hD1., 9Ou., 9Ou1., 9Ou2., 9Ou3., 9Ou4., 9Ou5., Xa0fZ, XaaBZ, XaaeA, XaaiW, Xabd2, Xabd3, XabEk, XabEl, XabtQ, XacIx, XacIy, XacIz, XacJ0, XacLx, Xacly, Xaclz, XacM2, Xaefu, XaJBQ, XaJBU, XaJBV, XaJBW, XaJBX, XaJPy, XaLFf, XaLFo, XaLFp, XaMFy, XaMG0, XaMGF, XaMGG, XaMGI, XaMGJ, XaMGK, XaMJC, XaYFR, XaYPX, XaZqJ, XaZWz |
| AD | Read V2 | Eu00., Eu000, Eu001, Eu002, Eu00z, F110., F1100, F1101, Fyu30 |
|  | Read CTV3 | .F21Z, Eu00., Eu000, Eu001, Eu002, Eu00z, F110., F1100, F1101, Fyu30, X002x, X002y, X002z, X0030, X0031, X0032, X0033, X003G, XaIKB, XaIKC, XE17j |
| VD | Read V2 | E004., E0040, E0041, E0042, E0043, E004z, E012., Eu01., Eu010, Eu011, Eu012, Eu013, Eu01y, Eu01z, F11x2, F21y2 |
|  | Read CTV3 | .E115, .E116, .G78., E004., E0040, E0041, E0042, E0043, E004z, Eu01., Eu010, Eu011, Eu012, Eu013, Eu01y, Eu01z, F11x2, F21y2, X003R, X003T, X003V, X003W, Xa0lH, XE1Xs |

Abbreviations: ACD, All-cause dementia; AD, Alzheimer's disease; VD, Vascular dementia; Read V2, Read codes version 2; Read CTV3, Read codes version 3.

Additional Figure 1. Estimated cumulative incidence curves with transplant related mortality (death) and dementia (ACD, AD, and VD ) as competing events for Model 1 scoring group (low risk (LR) and high risk (HR))


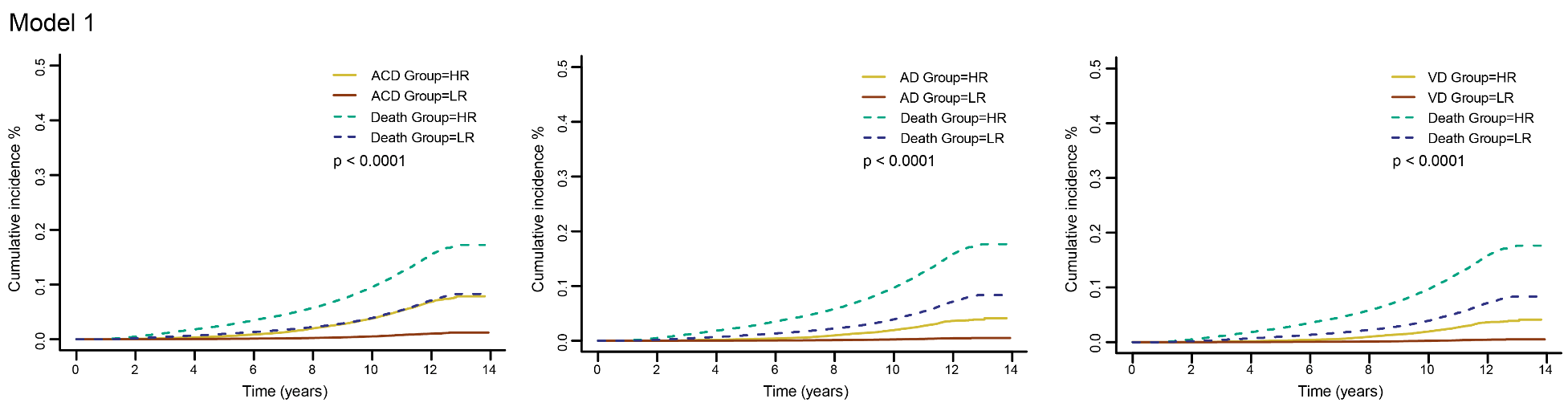


Additional Figure 2. Estimated cumulative incidence curves with transplant related mortality (death) and dementia (ACD, AD, and VD ) as competing events for Model 2 scoring group (low risk (LR) and high risk (HR))


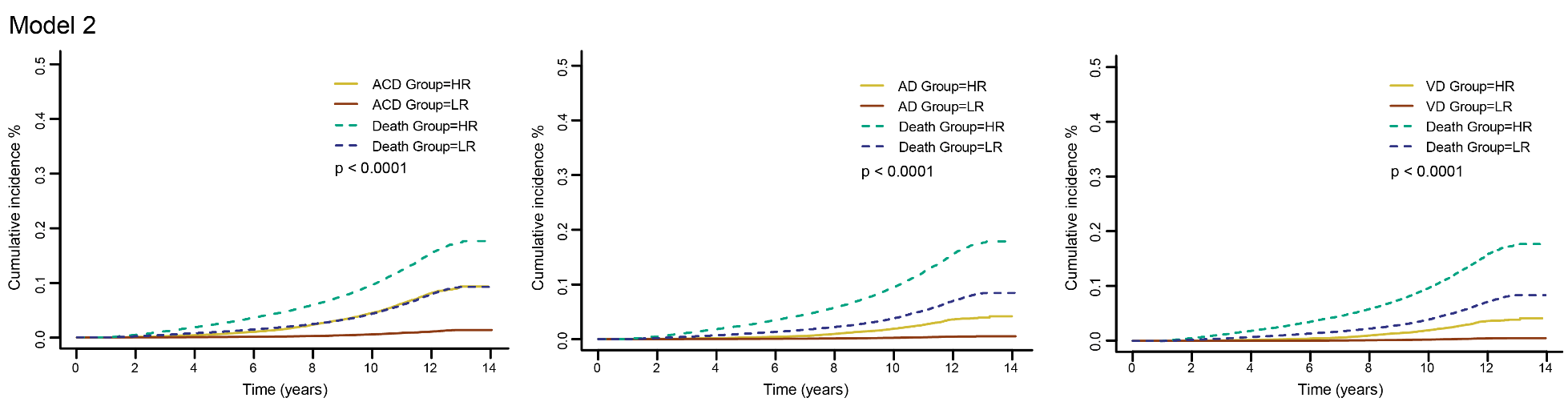

Supplement: Supplementary file 1 — Supplemental Information [file 41398_2022_2269_MOESM1_ESM.docx]
